# From Permits to Samples: Addressing Key Challenges for High‐Quality Reference Genome Generation in Europe

**DOI:** 10.1111/1755-0998.70100

**Published:** 2026-01-20

**Authors:** Katja Reichel, Jaakko Pohjoismäki, Jonas J. Astrin, Astrid Böhne, Chiara Bortoluzzi, Elena Bužan, Javier del Campo, Claudio Ciofi, Camilla B. Di‐Nizo, Pradeep K. Divakar, Carola Greve, Vladimír Hampl, Leon Hilgers, Veronika N. Laine, Jennifer A. Leonard, Jesus Lozano‐Fernandez, Lada Lukić Bilela, Camila J. Mazzoni, Ann M. McCartney, José Melo‐Ferreira, Rita Monteiro, Rebekah A. Oomen, Martina Pavlek, João Pimenta, Michal Rindos, Ole Seehausen, Andrii Tarieiev, Salvatore Tomasello, Olga Vinnere Pettersson, Robert M. Waterhouse, Alexandra A.‐T. Weber, Oleksandr Zinenko, Christian de Guttry

**Affiliations:** ^1^ Institute of Biology, Dahlem Center of Plant Sciences Freie Universität Berlin Berlin Germany; ^2^ Department of Environmental and Biological Sciences University of Eastern Finland Joensuu Finland; ^3^ Museum Koenig Bonn LIB Leibniz Institute for the Analysis of Biodiversity Change Bonn Germany; ^4^ Department of Biology University of Florence Sesto Fiorentino (FI) Italy; ^5^ Department of Aquatic Ecology Swiss Federal Institute of Aquatic Science and Technology (Eawag) Dübendorf Switzerland; ^6^ Environmental Bioinformatics Group SIB Swiss Institute of Bioinformatics Lausanne Switzerland; ^7^ Faculty of Mathematics, Natural Sciences and Information Technologies University of Primorska Koper Slovenia; ^8^ Faculty of Environmental Protection Velenje Slovenia; ^9^ Institut de Biologia Evolutiva, Biodiversity Program Universitat Pompeu Fabra Barcelona Spain; ^10^ Faculty of Pharmacy, Department of Pharmacology, Pharmacognosy and Botany Complutense University of Madrid Madrid Spain; ^11^ LOEWE Centre for Translational Biodiversity Genomics (LOEWE‐TBG) Frankfurt am Main Germany; ^12^ Senckenberg Research Institute Frankfurt Germany; ^13^ Faculty of Science, Department of Parasitology, BIOCEV Charles University Prague Czech Republic; ^14^ Faculty of Biosciences, Institute of Cell Biology and Neuroscience Goethe University Frankfurt Frankfurt Germany; ^15^ Finnish Museum of Natural History University of Helsinki Helsinki Finland; ^16^ Conservation and Evolutionary Genetics Group Estación Biológica de Doñana (EBD‐CSIC) Seville Spain; ^17^ Facultat de Biologia, Departament de Genètica, Microbiologia i Estadística Universitat de Barcelona (UB) Barcelona Spain; ^18^ Institut de Recerca de la Biodiversitat (IRBio) Universitat de Barcelona (UB) Barcelona Spain; ^19^ Faculty of Science, Department of Biology University of Sarajevo Sarajevo Bosnia and Herzegovina; ^20^ Biospeleological Society in Bosnia and Herzegovina Sarajevo Bosnia and Herzegovina; ^21^ Leibniz Institut für Zoo‐ und Wildtierforschung Berlin Germany; ^22^ Berlin Center for Genomics in Biodiversity Research Berlin Germany; ^23^ Genomics Institute University of California Santa Cruz California USA; ^24^ Departamento de Biologia, Faculdade de Ciências Universidade do Porto Porto Portugal; ^25^ Centro de Investigação em Biodiversidade e Recursos Genéticos (CIBIO), InBIO Laboratório Associado Universidade do Porto Vairão Portugal; ^26^ BIOPOLIS Program in Genomics, Biodiversity and Land Planning CIBIO Vairão Portugal; ^27^ Department of Biological Sciences University of New Brunswick Saint John New Brunswick Canada; ^28^ Tjärnö Marine Laboratory University of Gothenburg Strömstad Sweden; ^29^ Centre for Coastal Research University of Agder Kristiansand Norway; ^30^ Centre for Ecological & Evolutionary Synthesis University of Oslo Oslo Norway; ^31^ Ruder Boskovic Institute Zagreb Croatia; ^32^ Croatian Biospeleological Society Zagreb Croatia; ^33^ Biology Centre of the Czech Academy of Sciences Institute of Entomology Ceske Budejovice Czech Republic; ^34^ Faculty of Forestry and Wood Sciences Czech University of Life Sciences Prague Czech Republic; ^35^ Department of Fish Ecology & Evolution Swiss Federal Institute of Aquatic Science and Technology (Eawag) Kastanienbaum Switzerland; ^36^ Institute of Ecology and Evolution, Department of Aquatic Ecology and Macroevolution University of Bern Bern Switzerland; ^37^ Büsgen‐Institute, Department of Forest Genetics and Forest Tree Breeding University of Göttingen Göttingen Germany; ^38^ Institute of Biology/Geobotany and Botanical Garden, Systematic Botany and Biodiversity Martin Luther University Halle‐Wittenberg Halle (Saale) Germany; ^39^ Department of Systematics, Biodiversity and Evolution of Plants (With Herbarium) University of Göttingen Göttingen Germany; ^40^ Uppsala Genome Center, Department of Immunology, Genetics and Pathology Uppsala University Uppsala Sweden; ^41^ National Genomics Infrastructure SciLifeLab Uppsala Sweden; ^42^ V. N. Karazin Kharkiv National University Kharkiv Ukraine

**Keywords:** DNA/RNA preservation, eukaryotes, European Reference Genome Atlas, genome sequencing, high molecular weight DNA, sampling design

## Abstract

High‐quality reference genome assemblies have become essential for deepening our understanding of biodiversity, yet obtaining them for many species remains surprisingly challenging. Drawing on experiences from the European Reference Genome Atlas (ERGA) community, we focus on permit and sample‐handling procedures leading up to nucleic acid sequencing, covering tasks such as ensuring ethical and legal compliance, verifying accurate species identification, maintaining sample integrity during transport, and isolating high‐quality DNA or nuclei. While many of the challenges and solutions we discuss are broadly relevant, our regulatory and logistical examples are primarily from Europe. By synthesising practical guidance, we highlight the crucial importance of taxonomic expertise, proper vouchering and biobanking, rigorous cold‐chain management or alternative preservation methods, and emphasise adherence to packaging and shipping requirements for biological materials. We showcase examples spanning diverse regions, taxa and source materials, which underscore the importance of context‐specific strategies and internationally harmonised protocols, particularly for metadata reporting. Our recommendations aim to support both small‐scale projects and large initiatives, directing collective efforts to facilitate efficient sampling, vouchering and sample processing for future genomic studies.

## 1 Introduction

High‐quality nuclear reference genomes, defined as chromosomally scaffolded assemblies, serve as genomic standards for their respective species (Blaxter et al. 2022). Such assemblies are foundational for a wide range of applications, including transcriptome data analysis (Martin and Wang 2011), insights into gene content and structural variation (Mérot et al. 2020; Nurk et al. 2022; Sudmant et al. 2010), understanding genome and chromosome evolution (Damas et al. 2022), and facilitating phylogenomic (Rick et al. 2024), population genomic (Thorburn et al. 2023), and conservation genomic research (Theissinger et al. 2023). As the pool of species with high‐quality reference genomes grows across regions and taxa, new opportunities are emerging in ecological, evolutionary, biodiversity, conservation and bioeconomic research (Formenti et al. 2022; Theissinger et al. 2023).

Large‐scale initiatives such as the Earth BioGenome Project (EBP) and its affiliated projects, including the Darwin Tree of Life (DToL), have already provided guidance on sample collection and processing, DNA barcoding for species identification, and metadata standards for reference genome projects (Lawniczak et al. 2022, 2025; Twyford et al. 2024; Howard et al. 2025). As the formally recognised European regional node of the EBP, the European Reference Genome Atlas (ERGA) is a community‐led consortium of researchers and institutions coordinating reference genome sampling, sequencing, and applications within this global network (McCartney et al. 2024; Blaxter et al. 2025). Like other regional nodes, ERGA must translate EBP‐wide recommendations into region‐specific solutions compatible with legislative, administrative and operational realities across heterogeneous national contexts.

Currently, achieving high‐quality chromosome‐level assemblies relies on long‐read sequencing technologies, such as PacBio HiFi or Oxford Nanopore Technologies (ONT), combined with high‐throughput Chromosome Conformation Capture (3C, e.g., Hi‐C, Omni‐C, pore‐C; Lieberman‐Aiden et al. 2009; Belaghzal et al. 2017) and, in some cases, short‐read data for error correction (Yamaguchi et al. 2021). Genome assembly is often complemented by sequencing of RNA for content annotation (e.g., short‐read RNA sequencing [RNA‐Seq] and long‐read isoform sequencing [Iso‐Seq]), ideally from the same specimen but, especially for small‐bodied species, frequently from carefully matched conspecific individuals or pooled tissues of multiple individuals from the same population. These methods demand fresh or well‐preserved material with intact nuclei and/or RNA, making sample collection and handling critical. The prevailing standard is to flash‐freeze samples in liquid nitrogen immediately after field collection, implying that measures for accurate species identification, either prior to or after freezing, must be implemented to prevent the need for sample thawing. This requires maintaining a continuous ultralow temperature cold chain (ULT, −70°C or below) from the collection site to the laboratory or safe cold storage, a significant logistical challenge, especially where reliable shipping services are unavailable (McCartney et al. 2024).

Subsequent extraction of high molecular weight (HMW) DNA requires tailored protocols for different taxa (Varma et al. 2007). Depending on the complexity of the optimization process or sequencing requirements, substantial quantities of biological material may be needed, posing an additional obstacle for small organisms. Beyond technical constraints, logistic and administrative steps must be addressed before sampling begins. Identifying and involving local field experts, taxonomic experts, scientific collections, and skilled technicians is crucial, especially if different steps of the process (e.g., collecting, identifying, sequencing) are to take place in different countries. Ethical and sustainable sampling practices, as well as the necessary permits, must also be established before sampling begins (McCartney et al. 2024).

**FIGURE 1:**
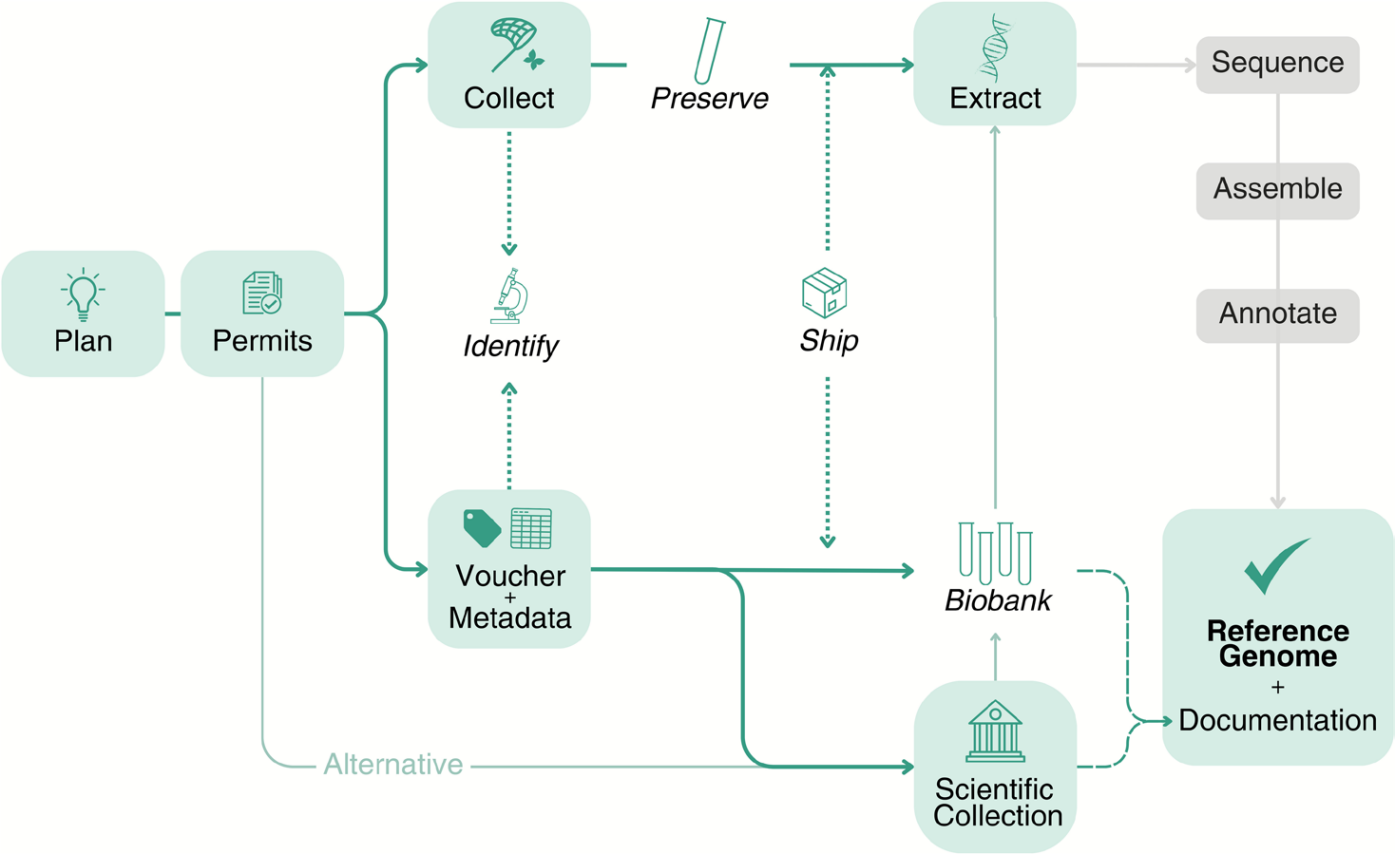
Overview of the workflow for reference genome generation. Steps in green boxes are discussed in this article; steps in italics with dotted arrows may occur in multiple places across the workflow. Thin arrows outline alternative routes to obtain sequenceable nucleic acids, whereas dashed arrows indicate information flow (voucher IDs, metadata). Illustration by Luísa Marins.

Building on global EBP and DToL guidance, we provide a transnational and multidisciplinary overview of best practices in sampling and sample handling for reference genome generation, covering permits, fieldwork, DNA isolation and biobanking, illustrated by case studies. Much of this guidance draws on practical experience gained during the first 3 years of the European Reference Genome Atlas initiative (ERGA, Mazzoni et al. 2023) and the Biodiversity Genomics Europe project (BGE, https://biodiversitygenomics.eu/), a Horizon Europe–funded programme piloting reference genome and barcoding workflows across European countries and institutions. At each major step, this overview is complemented by experience from individual cases, illustrating the range of challenges in large‐scale species‐level reference genome sampling across countries, remote or difficult‐to‐access locations, and non‐model or delicate organisms. Together, these examples highlight the importance of careful planning, strong collaborative networks, and an adaptable mindset. They also underscore that no single approach covers all cases: each organism and situation demands its own strategies to ensure the successful capture and preservation of high‐quality genetic resources and its correct taxonomic attribution, although common obstacles, best‐practice standards and objectives can be described. This guide follows the typical sequence of a reference genome project (Figure 1), from upstream ethical considerations and permits, through field sampling and preservation, to downstream nucleic‐acid extraction and complementary approaches, such as museomics.

## 2 Ethics and Permits

Reference genome sequencing typically involves the collection and handling of biological material, sometimes even from species that are protected or hazardous. Therefore, ethical, legal and administrative issues must be carefully considered long before any sampling is conducted. When specimens or extracted materials must be transported across international borders in order to be sent to laboratories or sequencing centres, the challenge is even greater. Planning well in advance and maintaining open, honest communication with local stakeholders is imperative in such circumstances.

The Convention on Biological Diversity (CBD 2011) establishes a framework for fair access and sharing of benefits, ensuring that countries retain control over their genetic resources. This has given rise to access and benefit‐sharing (ABS) frameworks, which regulate how genetic resources can be accessed and used, and how resulting benefits are shared between provider and user countries. In many jurisdictions, these ABS obligations are implemented through the ‘Nagoya Protocol on Access to Genetic Resources and the Fair and Equitable Sharing of Benefits Arising from their Utilization’ and its national implementing measures, which may require formal Nagoya‐related access permits, including Prior Informed Consent (PIC) and Mutually Agreed Terms (MAT), before collecting or exporting specimens for genomic work. Essentially, these agreements were established to prevent the repetition of past misuses of biodiversity and to promote responsible, equitable research (Nehring 2022). Additionally, regional measures such as the Bern Convention (ETS 104 1979), the EU Council Regulation on Trade in Wild Fauna and Flora (EC 338/97 1996), the International Treaty on Plant Genetic Resources for Food and Agriculture (ITPGRFA 2009), the United Nations Convention on the Law of the Sea (UNCLOS 1982), and the Biodiversity Beyond National Jurisdiction (BBNJ 2023) agreement can also play important roles in guiding reference genome projects in Europe (McCartney et al. 2024). Comparable regional and national frameworks operate in other parts of the world, but a detailed treatment of those lies beyond the scope of this Europe‐focused overview.

Global agreements often require domestic legislation to translate their provisions into enforceable actions. Consequently, regional directives, such as the EU Birds and Habitats Directives (92/43/EEC 1992; 2009/147/EC 2009), prohibit the capture, keeping, sale, transport, or killing of protected species, and likewise forbid disturbing them or destroying their habitats. Researchers may need to secure permits for sampling protected taxa, but also for accessing restricted sites such as national or regional parks. Moreover, import/export certifications (e.g., phytosanitary or veterinary permits) commonly apply when biological material crosses national or subnational borders, each regulated by different authorities. These processes often involve country‐ and region‐specific regulations, so planning ahead and contacting relevant agencies early can mitigate delays.

Determining which laws or regulations apply to a particular species or habitat, identifying required permits, and understanding how to obtain them can be challenging, particularly for those working outside their home countries. Language barriers, the varied implementation of international treaties, and regional legal complexities add to the difficulty (Box 1). To address these issues, researchers can rely on:

1. Dedicated platforms such as the Access and Benefit‐Sharing Clearing‐House (ABSCH, https://absch.cbd.int) under the CBD Secretariat, which consolidates national ABS measures, permits and contact points, and offers guidance on country‐specific requirements.
2. The Convention on International Trade in Endangered Species of Wild Fauna and Flora Database (https://checklist.cites.org), an authoritative reference for species regulated under CITES (CITES 1973), including trade restrictions and required documentation.
3. National or regional websites detailing specific rules, from Competent National Authorities and National Focal Points to checklists of official forms.
4. NGOs, scientific collections, professional societies, or research networks with practical experience navigating ethical frameworks

In many regions, local communities and Indigenous peoples hold long‐standing relationships with native species and ecosystems. Consultation or partnership with these groups may be required under local customary law or broader ethical guidelines. Partnership is also good practice to ensure that sampling, data interpretation, and publication respect community rights, cultural practices, and traditional knowledge. Practical starting points include identifying relevant community bodies (e.g., tribal or local councils, Indigenous organisations or community conservation groups), seeking advice from in‐country partners on appropriate contacts, and allocating time and budget for meetings before sampling begins and for meetings after sequencing and analysis are complete for insights to be shared. Early conversations should clarify research goals, potential risks and benefits, expectations around recognition, data access and use, and how results and non‐monetary benefits will be returned. Where available, researchers should follow established Indigenous research ethics and data‐governance frameworks such as the Collective benefit, Authority to control, Responsibility and Ethics (CARE) Principles (Carroll et al. 2020) and discipline‐specific guidance for genomic research with Indigenous communities (Claw et al. 2018). Even where formal procedures are not legally mandated, building relationships based on transparency and reciprocity should become standard leading to more equitable partnerships and more robust science.

BOX 1 **Experience: Permits in Bosnia and Herzegovina, Ukraine, Slovenia and Germany.**

This box illustrates how different national and subnational permitting systems, even within Europe, require early engagement with local authorities and in‐country researchers, and how ERGA partners have navigated these differences in practice.

**Bosnia and Herzegovina** is exceptionally biodiverse, harbouring an estimated 5000 vascular plant species, over 10% of which are endemic, and a rich vertebrate fauna, with 39 species endemic to the region (Redžić et al. 2008). The country is also particularly renowned for its rich subterranean fauna (Culver and Pipan 2013; Delić et al. 2023), which has historically made it an international target for the illegal extraction and use of its biological material from subterranean ecosystems (Lukić Bilela and Jelić 2018). While the country has ratified the CBD, it has not yet signed the Nagoya Protocol but enforces its environmental regulations. Nature protection legislation is managed separately in the Federation of Bosnia and Herzegovina (FBiH), the Republic of Srpska, and the Brčko District. The FBiH enacted its updated Law on Nature Protection in 2013 (‘Službene novine Federacije BiH’, No. 66/13, 10/25), addressing biodiversity conservation, habitat classification, species protection, and the implementation of the Natura 2000 network in line with EU directives. Similarly, the Republika Srpska has its own Law on Nature Protection (‘Službeni glasnik Republike Srpske’, No. 49/2024), harmonised with international frameworks such as the EU Habitats and Birds Directives. The Brčko District follows a harmonised approach that aligns with other entities to ensure cohesive environmental management (‘Službeni glasnik Brčko distrikta BiH’, No. 16/2024). Together, these laws reflect the country's commitment to biodiversity conservation, public participation, and international standards. Local and foreign researchers therefore need to engage directly with permitting authorities and local partners to ensure that sampling complies with these regional laws.

**Ukraine** recently increased its participation in international research projects aimed at describing biodiversity using genomic data. It ratified the Nagoya Protocol in 2022, a few days before the onset of de‐facto war,^1^ and thus did not implement any further legislative measures to ensure the sustainable use of its natural resources and prevent biodiversity loss, except for those already in force. Its policies do not currently address benefit‐sharing mechanisms related to the use of genetic resources, neither domestically nor internationally (Godlevska et al. 2018; Law of Ukraine No. 2818 2010). Due to the ongoing armed conflict (here defined as in International Committee of the Red Cross 2024), the necessary infrastructure for issuing permits, clearing approvals for the transfer of genetic material, and following the administrative procedures required by the Nagoya Protocol has not been fully established (www.cbd.int/countries/?country=ua). As a consequence, most research under these extraordinary circumstances does not require otherwise compulsory permits. Sampling and sample handling must be authorised by the Ministry of Environmental Protection and Natural Resources of Ukraine (MEPR); however, a formal process for issuing permits exists only for protected areas, such as national parks and nature reserves, and for species listed in the Red Book of Ukraine, which consists of two separate volumes for animals (Akimov 2009) and plants (Didukh 2009b), and plant communities listed in the Green Book of Ukraine (Didukh 2009a). This leaves the majority of species unregulated, creating a legal vacuum regarding access to genetic resources. Shipping biological material through international delivery services presents further challenges, as many major courier companies have ceased operations in Ukraine since 2022. Those still operating often lack experience and must navigate a regulatory grey zone without clear guidelines. Additional obstacles include customs declarations and veterinary certification requirements for exporting biological samples from Ukraine to the EU. These logistical and regulatory barriers, together with the absence of research infrastructure within the country, hinder genomic biodiversity research in Ukraine. Addressing these challenges will require harmonising legislation, developing clear administrative procedures, and building the necessary infrastructure to support research and international collaboration.

**Slovenia** is home to the ERGA national node ERGA.SI, where eight Slovenian research institutions engaging in biodiversity genomics have united to request collective collection permits for species identified as priorities for reference genomes in the BGE project. From the outset, they have been collaborating closely with the country's key permitting authorities, the Ministry of Agriculture, Forestry and Food, and the Ministry of Natural Resources and Spatial Planning, to streamline the permitting process and reduce approval times. Slovenia complies with the second part of the Nagoya Protocol, which governs the obligations of users of genetic resources, as prescribed by Regulation (EU) No. 511/2014 for all EU Member States and implemented through Slovenia's national regulation. Currently, obtaining and transferring the specified material does not require prior informed consent (PIC) and mutually agreed terms (MAT), but Slovenia has established biobank‐based procedures for granting access to its genetic resources within the EU ABS framework. In practice, ERGA.SI has become a key intermediary, guiding sample collectors through both national and international permitting processes, and liaising directly with ministries when no ethical permit is needed. This approach has not only simplified the permitting process but also ensured that researchers meet all legal and ethical obligations. The Slovenian experience shows that a dedicated, locally embedded consortium like ERGA.SI can serve as an effective central point of contact between researchers and administrative bodies supporting biodiversity research.

In **Germany**, the Federal Agency for Nature Conservation (BfN) is designated as the Competent National Authority for the Nagoya Protocol and is equally responsible for enforcing CITES regulations. It maintains an online database (https://www.wisia.de) that lists species protected at the federal or international level. However, the BfN does not issue sampling permits and does not inform on additional state or district laws further restricting the sampling of particular taxa (e.g., fish) or access to nature reserves. For example, collecting a protected plant species for genome sequencing from a single location for the BGE project required exemptions under both national and EU legislation, as well as permission to access a protected area. These permits can usually be obtained from a single authority, but the responsible agency and administrative tier (district or state) varies between Germany's federal states. Moreover, local conservation managers may have to be informed in advance, and all correspondence and documentation must be conducted in German, underscoring the importance of engaging local collaborators who can assist with both language and bureaucratic nuances. Having a well‐informed local collaboration partner is therefore invaluable, ensuring that communication is effective, documentation is complete, and the necessary permits are obtained without unnecessary delays.

***Take home message:*** treat permitting as a multi‐layered process (national, regional, protected areas) and identify the competent authorities well before fieldwork. Whenever possible, work with in‐country researchers or institutions, as they are essential for interpreting local law, language, and administrative culture as well as for interpreting downstream results in local context. Document permit workflows and share templates within consortia, so that subsequent projects can build on proven routes rather than starting from scratch.

## 3 Sample and Metadata Collection

Once the sampling location is defined and the relevant permits have been obtained or are in sight, the sampling campaign itself requires careful planning to avoid unnecessary harm to the species in question or its environment, or additional costs for repeated trips due to samples not meeting the minimum requirements. The key steps are field collection, specimen and tissue vouchering, documentation including metadata collection, taxonomic identification, and sample preservation. Different circumstances may call for different approaches: preserving tissue or blood by flash‐freezing it in remote or difficult‐to‐access areas will require additional planning and other equipment than bringing a whole organism alive to the lab for further processing. Sampling from already‐collected material stored in natural history collections or other genomic resource collections can substantially reduce these challenges, provided the material was preserved in ways compatible with downstream genome sequencing. This special case is treated separately further down, while this section pertains to sampling from the wild or from living collections (e.g., zoos, botanic gardens). The recommendations in this section align with the ‘before, during, and after fieldwork’ phases summarised as a checklist in Data S1.

As a general rule, the main tenets of ensuring that high‐quality, well‐documented genetic material is brought to the lab are: (1) keeping tissues or cells alive for as long as possible, (2) maintaining a continuous cold chain for preserved samples, (3) verifying species identity as early as possible (ideally before sacrificing/freezing), and (4) collecting as many types of vouchers (specimen, tissue, DNA, pictures) and metadata (time, coordinates, site description, environmental conditions, and other contextual information) using standardised formats (e.g., ERGA Manifest, Darwin Core, Minimum Information about any [x] Sequence [MIxS]) as possible. By using this approach, not only is the integrity of the biological material preserved, but also a rich contextual framework for future research is provided, enabling reproducibility and facilitating data reuse (Böhne et al. 2024). This applies equally to ‘easy‐to‐sample and easy‐to‐process’ target species and to more challenging taxa that are small‐bodied, rare, cryptic, or logistically difficult to access, which we illustrate through the case studies on cave fauna, cell cultures, single‐celled eukaryotes and museomics (Box 2).

In many reference genome projects, permits limit the number of individuals that can be sampled, restrict sampling to non‐lethal methods (e.g., only blood from endangered birds or mammals), or cap the amount of tissue that may be removed. Under such conditions, experiment design and sample use must be prioritised prior to fieldwork. A practical approach is to define a specimen‐ and tissue‐allocation plan in advance, specifying which assays are essential (e.g., HMW DNA, 3C, RNA for annotation, barcoding, voucher tissues, photographs) and how they will be derived from each specimen/sample. Wherever possible, samples should be collected so that a single capture event supports multiple downstream workflows, for example by separating whole blood into fractions that provide both HMW DNA and viable cells, or by pairing a small, non‐lethal biopsy with high‐quality whole‐organism photographs and environmental metadata. When sample numbers are strictly limited, projects should coordinate to avoid competing uses of the same material, and laboratories should pilot extraction and library protocols on non‐critical material before using rare samples. Close coordination with museums and biobanks during planning helps ensure that the small number of permitted specimens is split optimally between genomic analyses, long‐term preservation, and morphological vouchering.

### 3.1 Collection, Handling Time, and Preservation

Nucleic acids, including the HMW DNA required for high‐quality assemblies and the RNA needed for functional annotation, degrade rapidly upon an organism's death, making quick and effective preservation a top priority (Dahn et al. 2022). In general, handling time prior to sample preservation must be minimised, and temperature maintained at ULT conditions (at or below −70°C), which are sufficient for long‐term storage of most biological samples and more energy‐efficient than operating at −80°C (Beekhof et al. 2012; Espinel‐Ingroff et al. 2004). For species that cannot be brought alive to a laboratory or which are too large to be collected whole, field preservation is essential: ideally, tissues should be flash‐frozen in liquid nitrogen (−196°C) or placed on dry ice (−78.5°C). Exceptions include e.g., blood samples, which may benefit from collection in ethylenediaminetetraacetic acid (EDTA)‐filled tubes or other specialised storage buffers that maintain sample quality until further processing. However, EDTA‐based preservation can affect histone integrity and is not compatible with all 3C protocols. Some workflows are more tolerant to EDTA than others, whereas certain commercial Hi‐C kits explicitly advise against EDTA‐preserved material. It is therefore important to specify the intended downstream applications (e.g., standard DNA sequencing only versus also Hi‐C or other 3C methods) before choosing a preservation buffer and to follow the recommendations of the chosen kit or protocol. Liquid nitrogen, typically transported in absorbent‐lined ‘dry shippers’, and dry ice in insulated containers require special care and handling skills (Gordy et al. 2019). Depending on container design, outside temperature and handling, these systems may maintain ULT for days to weeks and can often be transported as luggage if they comply with airline regulations such as the International Air Transport Association's (IATA) Special Provision A152. However, they still come with logistical and regulatory constraints, and their effectiveness depends on maintaining cryogenic temperatures throughout transport; if dry ice or liquid nitrogen is not replenished during extended transit, temperatures may rise quickly and compromise sample integrity.

**FIGURE 2:**
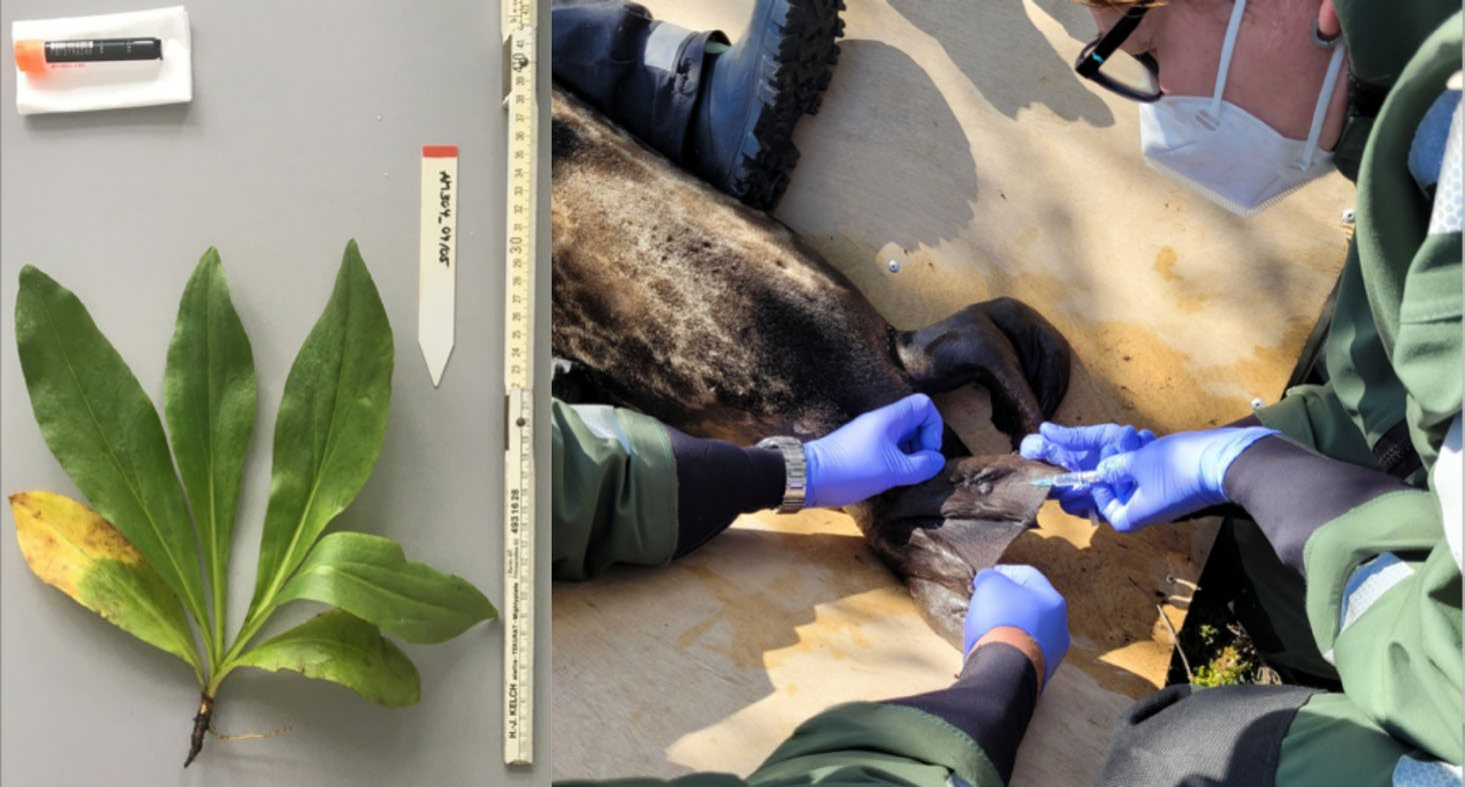
Tissue sampling in the laboratory and the field. Left: the medicinal plant mountain arnica ( *Arnica montana* L.) was preserved in the laboratory. Since this specimen was used up for tissue sampling and does not include all organs with characteristic features for the species, a flowering specimen from the same clonal network was deposited as a proxy voucher in the Berlin herbarium. Photo by Katja Reichel. Right: minimally invasive sampling of the endangered Saimaa ringed seal (*Pusa saimensis* [Nordquist, 1899]) during a relocation project. A small skin sample of the hind flipper was dissected under local anaesthesia and placed in a collection tube with cell culture medium; a blood sample was taken, and the animal measured. Photo by Mikko Suonio, Metsähallitus, with permission.

Where such low‐temperature conditions are not feasible, alternative approaches may delay nucleic acid degradation. For many animal taxa (vertebrates and invertebrates), tissues instantly submerged in 96% ethanol at a minimum ratio of 1 part tissue to 10 parts ethanol (*v*/*v*) and subsequently kept refrigerated for up to 7 days can still yield DNA of high integrity (Dahn et al. 2022; Marquina et al. 2021). Where possible, replacing the ethanol after the first 1–3 days helps to counteract dilution by tissue‐derived water and maintain a high effective alcohol concentration. Biopsies of viable tissue kept in cell growth media and antibiotics at 4°C for subsequent cell culturing (Wong et al. 2012) or transporting live organisms (e.g., seeds and bulbs) may be possible for certain taxa (Figure 2). For RNA preservation, reagents such as RNAlater or TRIzol are widely used (Camacho‐Sanchez et al. 2013), but TRIzol is toxic and volatile, so its use and transport in the field requires appropriate personal protective equipment (including eye protection and, where ventilation is poor, respiratory protection), careful labeling and disposal, and compliance with local regulations on hazardous chemicals. For field teams without extensive molecular‐lab experience or dedicated chemical‐safety infrastructure, we therefore recommend favoring non‐hazardous preservation solutions such as RNAlater or similar buffers and reserving TRIzol‐based workflows for controlled laboratory settings. Newer N,N‐dimethylacetamide‐based products (e.g., AllProtect, DNA‐Guard) offer partial DNA protection but can affect downstream applications like 3C (Dahn et al. 2022).

Even though live material is often ideal for extracting high‐quality DNA/RNA, at least part of the specimen should still be preserved as a tissue voucher, making sample preservation unavoidable in genome generation projects. Where this is not possible (e.g., very small organisms that are completely used for extraction), one or more additional individuals from the same species, population and sampling event should be preserved as a proxy voucher, that is, one or more additional individuals from the same species, population and sampling event that stand in for the sequenced individual (Pleijel et al. 2008). In the laboratory, the standard approach is to dissect the cleaned and reliably identified organism (if necessary) on a clean, cooled surface, such as a glass or metal plate on (dry) ice or liquid nitrogen, then collect the tissue in pre‐labelled and pre‐chilled tubes and flash‐freeze it in liquid nitrogen using long tweezers, wearing thermogloves, long sleeves, and protective goggles. For plants, algae, and fungi, chilling the dissecting surface can be counterproductive if it causes wilting before flash‐freezing. Instead, the priority is to keep tissues turgid as long as possible. Care should be taken to avoid mixing up samples: taking precise notes or, better still, a photo of each tube label and contents before freezing, along with recording the coordinate of each sample in the storage box, can prevent confusion if labels become illegible in the freezer. Additionally, using tubes and labels specifically designed for cryogenic conditions is important, as not all materials are resistant to extreme temperatures and may degrade or peel. Once flash‐frozen, samples must be carried on dry ice and stored at ULT until further processing. In the field, this procedure must be adapted to logistical constraints, again often precluding the use of sophisticated laboratory equipment or reliable cold‐chain storage.

### 3.2 Species Identification

Accurate identification of the collected species is fundamental to the utility of its genome sequence (Kürzel et al. 2022). Ideally, identification is performed by a qualified specialist, where possible in the field, although morphological examination in the laboratory is often necessary to distinguish small morphological characters. For material from botanic gardens, sexual offspring of plants not collected in the wild should typically be excluded from reference genome sequencing, owing to the high risk of hybridization. The same caution applies to other taxa where hybridization may be an issue, such as many species of fish from the aquarium trade, as well as to captive individuals of unknown provenance.

Apart from ‘connecting’ separate specimens, some of which are used for genome sequencing while others are deposited in collections as morphological vouchers, DNA barcoding may serve as an additional line of evidence for taxonomic identification (Chorlton 2024; Federhen 2015; Meiklejohn et al. 2019), especially for cryptic taxa, although DNA barcoding may not identify samples to species level in closely related taxa. Within the DToL project, DNA barcoding has been formalised into a standardised framework for species identification and sample triage (Twyford et al. 2024), providing a practical template for integrating barcodes into reference genome workflows that ERGA and other regional nodes can adapt to their own contexts. However, barcoding must be interpreted with caution, as organellar markers have low resolution and can usually not distinguish species that are younger than a million years, and/or may have introgressed between species that are even considerably older and belong to distinct genera. Appropriate markers vary with taxon (Hollingsworth 2011; Pawlowski and Holzmann 2014; Schoch et al. 2012) and a taxon specialist may be able to contribute to this choice as well. However, for certain taxonomic groups no specialist may be available, highlighting the continued need for trained taxonomists in the age of reference genomes, as well as the importance of depositing voucher specimens in recognised collections for future verification and study. Wherever feasible, we recommend combining expert morphological identification with standardised DNA‐barcoding workflows, using taxon‐appropriate markers, photographs, and tissue types and depositing barcode sequences in public repositories (e.g., the Barcode of Life Data System, BOLD, or the International Nucleotide Sequence Database Collaboration, INSDC). These project‐level barcoding SOPs provide step‐by‐step guidance that individual laboratories can adapt to their own taxa and infrastructure.

### 3.3 Vouchering, Biobanking, and Metadata Collection

**FIGURE 3:**
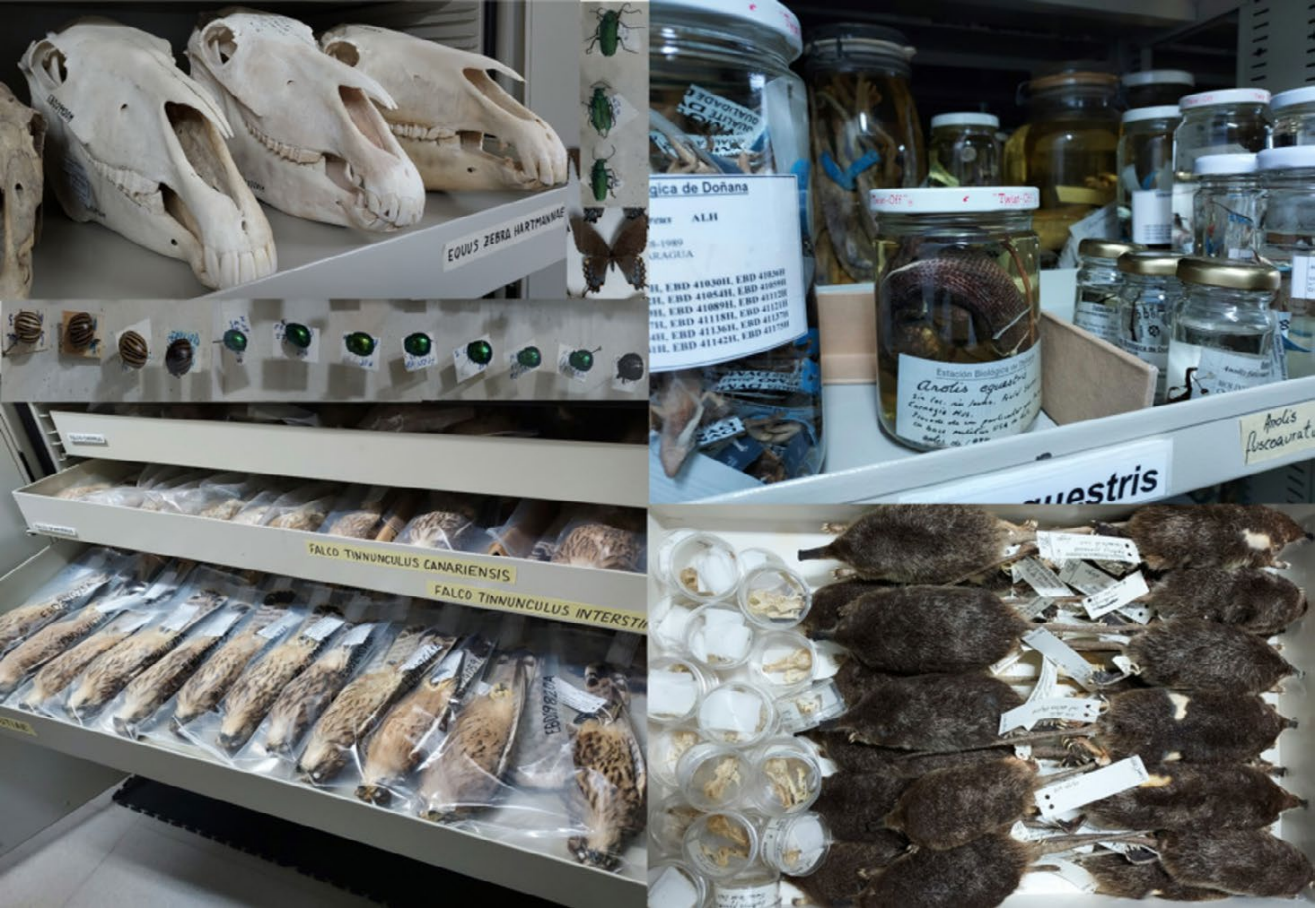
Animal vouchers in the natural history collection of the Estación Biológica de Doñana. Photos by Jennifer Leonard.

To maximise the value of a high‐quality genome sequence and to establish it as a long‐term reference, it should be complemented with appropriate metadata and vouchers. This principle has been emphasised in recent syntheses on the role of vouchers in genomics and open science (e.g., Schilthuizen et al. 2015; Buckner et al. 2021), and our recommendations build on this work in the specific context of reference genome sampling. Vouchers can vary in appearance depending on the taxon sampled, but should always include a physical specimen and, wherever feasible, a photographic image of the entire organism, ideally taken alive to capture natural coloration and morphology (Figure 3). Ideally, sufficient material remains after tissue sampling for genome sequencing, so that a whole‐organism morphological voucher can still be deposited. However, some taxa pose vouchering challenges due to their extremely large or very small size, or because they have soft or amorphous tissues. Large vertebrates, for example, may only be represented in collections by parts such as skeletons or skulls, whereas very large plants are represented by branches with leaves and reproductive structures. Conversely, if specimens are too small or damaged, additional samples from the same species and collecting event, or at least from the same locality, identified by the same taxonomist and ideally genetically related (e.g., from the same clonal family or fungal/lichen thalli in close proximity) may serve as proxy vouchers.

A tissue sample for future genetic studies should also be preserved in association with the morphological voucher and genome (Astrin et al. 2013). Ideally, these are flash‐frozen, viable cells already preserved along with the material for sequencing, but samples of lower quality, for example, in 96% ethanol or silica‐dried for plants, may serve as a substitute. To render vouchers permanently findable and publicly accessible, they must be deposited in a scientific collection under a stable identifier (typically including the institute acronym, collection acronym, and collection number), referenced in publications and future research. However, multiple preparations of a single specimen (e.g., dissected or separately stored parts, images, and associated sequence data) will often get separate labels and identifiers. Recording and cross‐referencing these identifiers is highly important for accurate documentation and traceability, especially as digitization efforts increasingly yield ‘digital specimens’, that is, online representations that complement physical vouchers (DiSSCoTech 2020).

During the permit application process, and thus well before fieldwork, it is advisable to consult with museum curators, collection managers, and taxon and field specialists to determine the most appropriate voucher materials for the taxon in question. While there are public lists for some types of museum collections, such as herbaria (Index herbariorum, https://sweetgum.nybg.org/science/ih/), others may only be found through general databases such as GenBank Biocollections (https://www.ncbi.nlm.nih.gov/biocollections) or the Global Registry of Scientific Collections (GRSciColl, https://scientific‐collections.gbif.org). As only a few (if any) institutions have staff dedicated to most taxonomic groups, engaging external taxonomists or experienced field researchers can fill this gap. Early communication ensures that vouchering requirements are addressed from the outset. This facilitates proper specimen preservation, storage, and documentation upon arrival in a museum or other scientific collection and actual value for taxonomic research.

Classic morphological vouchers include: pinned or ethanol/formalin‐preserved specimens for invertebrates, whole fishes and amphibians fixed in formalin and stored in ethanol, and study skins, skeletons or mounted specimens for birds and mammals; herbarium sheets or capsules for plants, fruiting bodies for macroscopic fungi, and dried agar cultures for microscopic fungi. Cultures or spore suspensions of culturable microscopic fungi, as well as cultures or long‐term cryopreserved stabilates of single‐celled eukaryotes, should be deposited in specialised culture collections findable through the European Culture Collections' Organisation (ECCO, https://www.eccosite.org/). Fixed specimens of single‐celled organisms, such as on microscope slides or as electron microscopy preparations, may also serve as valuable morphological vouchers, even though, so far, no universal repository systematically archives these materials. Cells, tissue or blood samples, as well as DNA/RNA extracts for future molecular work, should be curated in a specialised biobank, preferably affiliated with the Global Genome Biodiversity Network (GGBN, https://www.ggbn.org/ggbn_portal/members/index).

Whenever possible, vouchers are best deposited near the region where they were collected. Photographs of the organism in its habitat, particularly if it is too large to collect in full or if its coloration is lost on preservation, taken before sampling or preservation, or highlighting microscopic features crucial to identification, further enhance the voucher's usefulness. Where logistic and ethical constraints allow, collecting duplicate specimens or subsamples makes it possible to lodge material both in local institutions in the country of origin and in international or specialist collections, increasing long‐term security, accessibility, and equity in the use of genomic resources. At the same time, each additional voucher or tissue sample carries real administrative and financial costs for the receiving collection. Many natural history museums and biobanks operate under chronic constraints in space, curatorial staff time, and operational funding, even as expectations of vouchering and digitisation increase (Schilthuizen et al. 2015; Astrin and Schubert 2017; Salvador and Cunha 2020). We therefore recommend that reference genome projects treat collection managers as full partners. Planning vouchering strategies with curators in advance, explicitly budgeting for accessioning, long‐term storage, and digitisation in grant applications, and, where possible, supporting local infrastructure and training. This helps ensure that calls for more comprehensive genomic vouchering follow hand in hand with sustainable support for the institutions that enable it.

While, in general, any available metadata should be recorded and retained, it is equally important to store it in standardised data formats to ensure interoperability. Preferences may vary between collections, yet common standards include the Darwin Core format (Darwin Core Task Group 2009), the ABCD standard (Access to Biological Collections Data Task Group 2005) for morphological data and the GGBN data standard (Droege et al. 2016) for molecular samples. The DToL project has demonstrated how such standards can be operationalised for biodiversity genomics through a specimen and sample metadata framework and associated sample manifest (Lawniczak et al. 2022), which ERGA and other EBP nodes are now building on. Building on these, the ERGA network has developed an open‐source metadata manifest that aligns key collection metadata, sample IDs, voucher stable identifiers, and permit documentation into a machine‐readable format (https://github.com/ERGA‐consortium/ERGA‐sample‐manifest). This manifest can be brokered via the Collaborative OPen Omics (COPO) platform (Shaw et al. 2024) to the INSDC, thereby ensuring metadata quality and indelibly linking each reference genome with its corresponding metadata and vouchers (Böhne et al. 2024).

### 3.4 Sample Shipping

Ideally, both DNA/RNA extraction and library preparation should take place in the same laboratory to minimise shipping of samples or extracted nucleic acids, thus reducing the risk of degradation and simplifying administrative processes. However, different facilities may be responsible for these steps. When transport becomes necessary, either to a sequencing facility or a specialised biobank, it is important to maintain a continuous cold chain. This poses challenges similar to the shipping of preserved specimens from the field to the laboratory, as dangerous goods such as dry ice may again be necessary. If transport in a dry shipper is not feasible or cost‐effective, the current best practice is to ship samples on dry ice in a sturdy styrofoam box (wall thickness at least 4 cm) with a loosely sealed lid, such as placing the styrofoam container inside a slightly larger cardboard box, to allow gaseous carbon dioxide to be released. A drop test from roughly 1.2 m high can be used to verify that the parcel remains sealed even when handled roughly. For large shipments, a minimum of 10 kg of dry ice (corresponding to a volume of at least 6.5 L inside the box) will generally maintain the contents at around −80°C for at least 48 h (Hafner et al. 2024). However, smaller shipments of up to a few hundred 2‐mL tubes may require considerably less dry ice (e.g., 4 kg) if properly insulated. Dry ice shipments, especially across borders, must include a UN1845/HAZMAT Class 9 label indicating the initial net weight of the dry ice, a ‘fragile’ label, and orientation arrows. Stating the expected delivery time frame is also advisable; avoid shipping over weekends or public holidays. Not all couriers offer dry ice shipping, as special training is required for handling these parcels, and dry ice in a parcel must always be declared. Some courier companies provide a dry ice top‐up service, which is invaluable if the parcel is delayed in transit, for example, due to customs control. It is therefore advisable to get in touch with the courier company in advance and specifically request this service, as filling out additional paperwork might be required.

Apart from the cooling agent, the samples themselves require adequate documentation to clear customs without delay or thawing. All necessary export/import permits (e.g., for CITES‐listed species) must be included, and protected species should be accompanied by the relevant collection permits. While some countries may require a commercial invoice or itemised statement confirming that the shipment has minimal commercial value, others allow a simple declaration of value that may only need to reflect shipping and insurance costs. In parts of the EU, additional declarations or fees may apply for packaging materials (e.g., plastic use or waste taxes), which should be clarified with local customs offices in advance. For shipments within the European Union, customs declarations are generally not required, but complete contact details for both sender and recipient (including phone numbers) should always be provided on the parcel and accompanying documents. For shipments entering or leaving the EU customs territory, a CN 23 customs declaration form and, where available, institutional tax or Economic Operators Registration and Identification (EORI) numbers are usually required, in line with national customs and postal regulations (Universal Postal Union and World Customs Organisation 2024). The Harmonised Commodity Description and Coding System (HS) codes typically used for scientific collection material fall under HS chapter 97 (e.g., HS 9705 2900). Unless specific regulations indicate otherwise, the content description should clearly state that the samples are non‐hazardous biological material for research use only and are not infectious or dangerous. Because regulations vary widely, especially between non‐EU countries, additional information or documentation may be required. Apart from museum curators, who usually engage in international scientific sample loans and shipping, local authorities (e.g., customs offices, permit‐issuing agencies) or specialised shipping services may also be able to offer guidance. Whenever possible, it is prudent to verify all documentation with the relevant regulatory bodies well in advance, to minimise the risk of delays or compliance issues at the border.

BOX 2 **Experience: Sampling Cave Dwellers, Vertebrate Cell Cultures and Single‐Celled Eukaryotes.**

This box highlights different strategies for technically challenging taxa: fragile cave species in difficult environments, vertebrates where lethal sampling is constrained, and single‐celled eukaryotes with limited biomass, illustrating how sampling, preservation and lab workflows must be tailored to biological and ethical constraints.

**Cave dwellers** not only include fascinating models for parallel evolution, as distinct taxa often converge on similar adaptations in lightless, low‐nutrient environments, but may also comprise unique relict lineages of major conservation value. The lightless, low‐nutrient and effectively isolated conditions in caves also make sampling cave dwellers a challenge. In the karstic regions of central and southern Europe, many surface‐dwelling species went extinct during Pleistocene glacial maxima, leaving cave habitats as refugia for ancient organisms. For example, the olm ( *Proteus anguinus* Laurenti, 1768), Europe's sole living proteid salamander, survives in subterranean waters across the Dinaric Karst (Recknagel et al. 2024; Recknagel and Trontelj 2022), while the freshwater amphipod genus *Niphargus* has undergone a remarkable radiation of hundreds of species extending from the Alps to Turkey and Greece (Biró et al. 2024; Borko et al. 2023). Yet, caves are frequently difficult to access, and their biota, often minuscule, delicate, and poorly studied taxonomically, can be challenging to collect and to keep alive. Transporting a dry shipper into a cave is typically impractical, especially in steep or vertical systems. However, for extremely fragile taxa such as palpigrades (Arachnida; ≤ 3 mm in length, prone to desiccation or drowning), direct collection into a barcoded tube and immediate storage in liquid nitrogen remains the best option.

Less sensitive cave‐dwellers can sometimes be transported alive to the laboratory. A low‐profile plastic container with approximately 2 cm of water‐saturated plaster at the bottom, potentially structured with shallow water‐filled hollows for isopods, keeps humidity high for weeks. More resilient insects, myriapods and arachnids may be fine in well‐sealed plastic tubes lined with moist tissue to prevent drying. For longer‐term storage, larger containers must be used and be aerated every few months. Aquatic cave‐dwellers travel best in containers two‐thirds filled with cave water, and frequent aeration, especially upon arrival in the lab, is advisable (Lukić et al. 2024). Because caves are usually cold, shipping temperatures should be kept comparable (e.g., via an electric cooler or an icebox with ice packs), ensuring animals are not placed directly on ice. Species identification in caves is often impractical due to low light and high humidity. However, identifying them alive in a lab environment also poses risks. Many cave systems have limited ecological niche diversity such that rarely more than one species per genus is found in a single cave, *Niphargus* amphipods and certain springtails being notable exceptions (Lukić et al. 2020; Trontelj et al. 2012). With thorough prior knowledge of a cave's fauna, teams can anticipate where multiple species are likely to co‐occur and focus their taxonomic checks accordingly, making species determination more straightforward once specimens are back in the lab.

**Vertebrate cell culture** is a highly useful technique to obtain material for genome sequencing while minimising the impact on the source individual. In most European countries, sampling vertebrate animals is usually subject to stricter legal and ethical controls than sampling invertebrates, particularly when invasive procedures or lethal collection are involved and may require additional permits. Being able to sample cells from live animals that are subsequently released again, or opportunistically from already dead individuals such as roadkill, is the best way to keep both bureaucratic effort and animal suffering as low as possible.

For certain taxa, blood sampling is straightforward because most fish, reptiles, amphibians, and birds have nucleated erythrocytes, facilitating easy extraction of high‐quality DNA (Delmore and Liedvogel 2020). However, mammals and very small‐bodied vertebrates lack nucleated red blood cells or pose additional logistical challenges. In these cases, cell culture can provide an excellent alternative for obtaining ample, intact DNA and RNA, particularly suitable for advanced protocols like Hi‐C (Belaghzal et al. 2017; Wong et al. 2012). This approach and its renewable source material also enable researchers to visualise karyotypes, investigate ploidy, or detect chromosomal anomalies (Deakin et al. 2019). Nonetheless, protocols for culturing non‐model species remain less common (Cardoso et al. 2024; Ezaz et al. 2008; Fekete et al. 2025; Michell et al. 2024; Mollard and Mahony 2023), and some trial and error may be needed to optimise conditions.

Establishing cell cultures typically requires minimal tissue, for instance, small biopsies of skin, eye tissue (birds and mammals), embryonic tissues (birds), ear or placenta (mammals), trachea (reptiles), or fins (fishes). Often, these samples can be collected as part of broader animal‐handling or fieldwork (Driskell and Watt 2015; Keane et al. 1979; Saski et al. 1968; Seluanov et al. 2010). Because microbial contamination is the main threat to successful culture, sterile technique is essential: small tissue segments, following 70% ethanol sterilisation, are transferred into cell media with fetal bovine serum (FBS) and antibiotics/antimycotics, in which they may remain viable for up to 2 weeks at 4°C, thus bypassing the need for a ULT cold chain. Once in the lab, fibroblasts are often the cell type of choice, although culture success depends on tissue characteristics (e.g., fat content, embryonic vs. adult cells) and taxa.

While cultured cells can be cryopreserved or biobanked (Ben‐Nun et al. 2011), it is important to note that mutations and chromosomal rearrangements can accumulate over successive passages (He et al. 2023) and, due to telomere shortening, cell lines eventually stop dividing (‘Hayflick limit’) (Shay and Wright 2000). If this is problematic, for example, when initial tissue is scarce, researchers may consider SV40 T‐antigen transformation (Bryan and Reddel 1994), which can extend lifespan without major genomic rearrangements (Adema 2021; Fekete et al. 2025; Michell et al. 2024; Turkalo et al. 2023). However, early‐passage cultures typically offer the most faithful representation of the genome, and any integrated vectors used for immortalization must be masked from the final assembly. In some cases, immortalised cultures in a growth medium can be shipped at room temperature, facilitating collaboration between multiple labs. Finally, as with all genomic resources, proper vouchering remains key: cell cultures or primary tissues should be unequivocally linked to vouchers just as any other molecular resource would be (see above). This ensures that genomic data can be reliably traced back to the individual organism, supporting reproducibility and broader comparative studies.

Single‐celled eukaryotes constitute a remarkably diverse group, varying greatly in morphology, physiology, and genome size, from just a few megabases to more than 100 gigabases (del Campo et al. 2014; Keeling and Slamovits 2005; Sibbald and Archibald 2017). Although approaches to single‐cell genomics continue to progress (Adema 2021; Mangot et al. 2017; Montoliu‐Nerin et al. 2020), obtaining a reference‐quality genome from a single cell using conventional workflows remains difficult, especially for those with larger genomes. Consequently, some form of amplification is usually required, either in vitro (e.g., through whole genome amplification, though error rates remain uncertain) or in vivo, by establishing a cell culture. Ideally, this means creating an axenic culture consisting of only a single species (or even the offspring of a single cell), but substantial effort is still needed to axenize a representative sample of the world's single‐celled eukaryote diversity (del Campo et al. 2024). Alternatively, some species can be cultivated in xenic culture, that is, alongside other microorganisms, or within a suitable host in the case of parasites and symbionts. In those instances, an organism‐specific purification step of the DNA (Aunin et al. 2021; Hampl et al. 2005) or a sophisticated bioinformatic filtering after sequencing (Zhao et al. 2024) is necessary, typically requiring prior knowledge of the host or other ‘contaminant’ genomes. For certain lineages, targeting specific life stages can circumvent some of these hurdles—for example, cyst stages that contain vast numbers of pseudoclonal cells can yield sufficient biological material without elaborate culturing (Boisard et al. 2022).

***Take home message:*** it is important to design collection methods around the habitat and physiology of fragile or logistically difficult taxa (e.g., cave fauna), rather than forcing ‘standard’ protocols to fit. Where lethal collection is not acceptable or feasible, (vertebrate) cell culture and blood sampling can provide high‐quality, renewable DNA and nuclei while minimising animal impact. For microscopic eukaryotes, plan from the outset whether you will rely on culturing, whole‐genome amplification, or life stages with many pseudoclonal cells, and anticipate the downstream need for contaminant removal.

## 4 Nucleic Acid Extraction

### 4.1 High Molecular Weight DNA

Although technical progress in sequencing continues apace, preparing the DNA or nuclei needed for high‐quality reference genomes can still be a formidable task, particularly for non‐model organisms and organisms with small body sizes. Unlike short‐read or polymerase chain reaction (PCR)‐based approaches, protocols for long‐read technologies currently demand ≥ 20 kb long, intact DNA molecules, while 3C methods require entire nuclei to remain intact. General care, such as using wide‐bore pipette tips, avoiding vigorous homogenization, and minimising repeated centrifugation at high centrifugal forces, helps to maintain molecular integrity. Commercially available kits (Table 1) can also facilitate the extraction of HMW DNA or nuclei, but these are often optimised for model species with abundant tissue. In many non‐model taxa, even standard ‘PCR‐grade’ methods, for example, a CTAB‐based approach, can yield adequately long DNA, though additional time and costs for method optimization should be anticipated. Researchers must also be prepared for inconsistent results among samples, even when using the same protocol on the same species. Detailed cross‐taxon wet‐lab workflows from large genome programmes, such as the Sanger Tree of Life core laboratory protocols (Howard et al. 2025), provide step‐by‐step examples that complement these general recommendations and can be adapted to local facilities and regulatory settings.

**TABLE 1:** Selection of commonly used protocols for HMW DNA extraction.

Name	Available from	Kit	Suggested for
Nanobind	PacBio	Yes	Animal tissue and cells, plant nuclei
Bionano Prep^a^	Bionano Genomics	Yes	Animal tissue/plant tissue versions
MagAttract	Qiagen	Yes	Animal tissue and cells
Genomic‐tip series	Qiagen	Yes	Animal tissue and cells
MegaLong	G‐Biosciences	Yes	Animal tissue and cells
Monarch HMW DNA extraction	New England Biolabs	Yes	Animal tissue and cells, insects
E.Z.N.A. Mollusc and Insect DNA	Omega Bio‐Tek	Yes	Molluscs, insects
NucleoBond	Macherey‐Nagel	Yes	Animal tissue and cells, plant tissue, fungi
Invisorb Spin Plant Mini	Invitek Diagnostics	Yes	Plant tissue
10x Genomics salting out (based on Miller et al. 1988)	10x Genomics Support (2017)	No	Animal tissue and cells, insects
Sanger Tree of Life Wet Laboratory Protocol Collection^‡^	protocols.io	No	Suite of protocols for various taxa
SPRI beads (Mayjonade et al. 2016)	protocols.io	No	Plant tissue, molluscs, versions tested for others
Phenol‐Chloroform (Sambrook and Russell 2006)	protocols.io	No	Animal tissue and cells
CTAB—many taxon‐adapted variants, usually coupled with Phenol‐Chloroform	protocols.io	No	Wide range of taxa, with taxon‐specific adaptations

*Note:* Information in the ‘Suggested for’ column is based on the statements of the protocol's authors _a_(also) nuclei extraction for 3C.

Typical challenges in HMW‐DNA extraction include mechanically disrupting tough cell walls in fungi, lichens, corals and various protists, or addressing taxon‐specific contaminants like oils, phenolics, resins, secondary metabolites, pigments and polysaccharides (Adema 2021; Varma et al. 2007). Preventing heat buildup—for example, by pre‐cooling grinding blocks and re‐freezing them with liquid nitrogen—can further reduce shearing. Specifically, using DNA‐rich tissues with fewer inhibitors may also help: testes or spleen often work well in vertebrates, whereas gills are preferable to the foot or mantle in molluscs, although those may contain parasitic organisms. Meristem‐rich young leaves, twigs or buds typically yield more DNA than older plant tissues, and have a much lower risk of contamination from other biota than roots. Transferring protocols from related species is often a good start, yet close phylogenetic relationships do not always translate to similar extraction performance, so pilot extractions are often necessary to identify suitable conditions.

Before sequencing, DNA quality is generally assessed via spectrophotometry (e.g., Nanodrop), fluorometry (e.g., Qubit or SYBR Green), and pulsed‐field gel electrophoresis (PFGE). Whereas Nanodrop or other spectrophotometry‐based instruments provide good information about the chemical purity of the sample, they are inferior for the assessment of DNA concentration when compared with fluorometric assays (Qubit or SYBR Green), which specifically measure the amount of double‐stranded DNA. Based on the empirical evidence from sequencing facilities within ERGA, more than 50% discrepancy in concentration measurement between spectrophotometry and fluorimetry is an indicator for carry‐over of contaminants, which can be detrimental to library preparation efficiency and compromise PacBio and ONT sequencing outcomes.

The integrity, or fragment length, of the extracted molecules is best assessed by Pulsed‐Field Gel Electrophoresis (PFGE), commonly seeking a minimum threshold of ≈20 kb for long‐read library preparation. Using Agilent FemtoPulse or FragmentAnalyzer is a good alternative to the traditional PFGE, as it helps to visualise fragments up to 165 kb in size, as well as to assess the share of degraded DNA in the sample (Dahn et al. 2022). Caution should be taken not to employ Agilent TapeStation for estimation of HMW‐DNA fragment length, as this instrument tends to overestimate length of molecules over 10 kb in size (Klingström et al. 2018). Even if the fragment length distribution metrics look promising, contaminants undetectable by conventional methods, single‐strand breaks, as well as DNA deamination or depurination can undermine sequencing output, or introduce sequencing artefacts, for example, erroneous single nucleotide polymorphisms (SNPs). Where unwanted compounds are suspected, amplification (Männer et al. 2024) or library clean‐up may offer a temporary fix, but severely compromised samples often require re‐extraction and resequencing. For many reference genome projects, perfecting nucleic acid extraction is an iterative process of trial, verification, and refinement.

To ease this optimisation, taxon‐ and tissue‐specific Standard Operating Procedures (SOPs) for HMW DNA are now being made available in open repositories. For example, the Sanger Tree of Life wet‐lab protocol collection on protocols.io provides validated extraction and nuclei‐preparation workflows for major eukaryote groups (Howard et al. 2025). Within BGE/ERGA, we are harmonising a complementary suite of sampling and extraction SOPs that are being released through the Biodiversity Genomics Europe/ERGA space on WorkFlowHub (https://workflowhub.eu/search?q=bge#sops) and protocols.io (https://www.protocols.io/search?q=dtol), covering both reference genome and barcoding use cases. These resources give practitioners concrete starting points, down to tube types, tissue amounts, and buffer compositions that can be adapted to local facilities and target taxa.

### 4.2 Nuclei Extraction for Chromosome Conformation Capture

Unlike standard long‐read sequencing, 3C workflows require intact nuclei to capture the three‐dimensional genome architecture, through crosslinking, fragmentation, ligation and pairwise short‐read sequencing of DNA stretches physically close to each other in the nucleus. While many fundamentals of HMW DNA isolation remain relevant, such as avoiding mechanical stress and preventing contamination, the emphasis shifts to preserving nuclear membrane integrity. Fresh or freshly flash‐frozen tissues (stored at ULT) typically provide better yield and quality than older or partially degraded specimens. Researchers usually rely on protocols for commercial kits, such as those offered by Arima Genomics or Dovetail, which start from cell suspensions (e.g., blood) or cryopreserved tissue and include specialised reagents for crosslinking, cell lysis, and DNA digestion/ligation steps. For blood and other liquid samples, the choice of collection buffer is therefore critical: preservatives with high EDTA concentrations can impair crosslinking and reduce Hi‐C signal in some 3C kits, so collection methods should be selected in line with the specific requirements of the intended workflow.

As with HMW DNA extraction, testing a few samples before committing large volumes of precious material can help to identify potential issues and optimal conditions. Microscopy or flow cytometry may help verify that the nuclei are intact, preventing wasted sequencing runs. Partial lysis or extensive mechanical disruption, particularly before the crosslinking step, can compromise nuclei integrity, thus reducing the 3C ‘proximity’ Hi‐C signal and complicating the genome assembly. The duration of the formaldehyde crosslinking reaction must be carefully timed to ensure adequate but not excessive linking, and cross‐linked material then cleaned and quantified prior to library preparation. Even within a given commercial kit, restriction digestion and shearing efficiency can vary with tissue type, cell composition and chromatin state. Accordingly, small pilot tests with different lysis and fragmentation conditions are often required. While kits streamline many steps, researchers should still adapt parameters to their specific taxa, tissues and storage conditions, as one‐size‐fits‐all solutions rarely apply in reference genome projects on non‐model organisms.

### 4.3 RNA Extraction

For reference genome projects, RNA sequencing is primarily used to support genome annotation by providing empirical evidence for exon–intron boundaries, splice junctions, untranslated regions and alternative isoforms, rather than for differential gene expression analyses. Nevertheless, once high‐quality libraries are available, the same datasets can also be used to explore expression patterns across tissues, conditions and life stages. Yet, securing high‐quality RNA for reference genome projects remains a delicate undertaking: unlike DNA, RNA degrades rapidly, making timeliness and rigorous cold‐chain management especially important. As contamination by DNA is another potential issue, RNA preparation should be performed apart from DNA preparation, or the workbench and tools thoroughly cleaned with DNAse in between. Standard laboratory protocols often rely on TRIzol or other guanidinium thiocyanate‐phenol‐chloroform reagents, which lyse cells efficiently and inactivate RNases; however, these involve toxic chemicals and multiple extraction steps, and must be handled with appropriate personal protective equipment, fume extraction and waste‐disposal procedures. For many reference genome projects—particularly those involving collaborators with limited molecular‐lab experience—commercial column‐based kits (e.g., from Qiagen or Thermo Fisher) provide a safer and simpler default, integrating lysis, on‐column DNase treatment, and silica membrane purification. Kits should be selected with care, preferring those which can retain multiple RNA types over those not capturing the miRNA fraction. As with DNA extraction, RNA kits and protocols may need testing and modification to accommodate non‐model organisms or atypical tissues. Although it introduces additional cost, time and optimization challenges, sampling RNA from multiple tissues or developmental phases of the same organism/species can significantly enhance genome annotation. Given the diversity of taxa and the rapid turnover of commercial kits, we do not attempt an exhaustive, kit‐level overview analogous to Table 1. Instead, we focus on general principles and decision points that laboratories can adapt to their specific organisms and infrastructure.

Preserving RNA integrity hinges on minimising delays between collection and stabilisation. Where liquid nitrogen or dry ice is available, flash‐freezing samples on‐site can be optimal; otherwise, RNAlater or NAP buffer, as well as homogenization of tissues in TRIzol reagent, provide a practical alternative under field conditions (Camacho‐Sanchez et al. 2013). These reagents, however, may not fully penetrate dense tissues and lower the total RNA yield. Once in the lab, immediate processing of unfrozen samples or storage at ULT is recommended. Persistent high DNA contamination, necessitating post‐extraction DNAse treatment, can be tested by fluorometry (e.g., Qubit comparing DNA and RNA assay). Verifying RNA quality through agarose gel electrophoresis or by inspecting electropherograms from an Agilent Bioanalyzer or Tapestation helps to detect degradation and contamination, both of which can diminish library performance or result in suboptimal sequencing. For vertebrates and other taxa with canonical 18S/28S rRNA profiles, a RNA Integrity Number (RIN) ≥ 8 is a useful rule of thumb for annotation‐grade RNA, but for many non‐model invertebrates and other groups with atypical rRNA patterns (e.g., arthropods, DeLeo et al. 2018; molluscs, Halstead‐Nussloch et al. 2024), RIN alone can be misleading. In those cases, we recommend focusing on the overall electropherogram profile (presence of high‐molecular‐weight RNA with limited low‐molecular‐weight smear) and, where possible, on pilot libraries and mapping statistics rather than rigid RIN cut‐offs. Where RNA sequencing is currently not feasible, at least appropriately preserved tissue samples (−70°C to −80°C, ideally separated by organ or life stage) should be kept so that RNA extraction and sequencing can be performed in future studies. For genome annotation, the choice of tissue is as important as the chemical quality of the RNA. For vertebrates, we typically prioritise transcriptionally rich organs such as the brain, liver and gonads. Where feasible, these tissues can be complemented by additional tissues that capture immune, sensory or developmental expression. For many invertebrates, whole‐body RNA can be supplemented with dissected thorax and abdomen or gonads. In plants, young leaves, other meristem‐rich tissues and reproductive structures, including seeds, usually yield diverse transcriptomes. These multi‐tissue panels, now routinely used in ERGA community genome projects, have markedly improved gene‐model completeness at a modest increase in sampling effort, and provide a practical default for future reference genome initiatives.

BOX 3 **Experience: Lab Methods for Lichenized Fungi and Marine Sea Slugs.**

This box provides examples of taxon‐specific biochemical and technical challenges, such as robust fungal cell walls, phenolic compounds, and mixed host–algal DNA in kleptoplastic sea slugs, and shows how modest protocol modifications can unlock high‐quality DNA from groups that are otherwise considered ‘difficult’.

**Lichenized fungi** remain taxonomically understudied (Niskanen et al. 2023), and generating HMW DNA from cultured mycobionts can be challenging. The first obstacle arises from their robust fungal cell wall, which is composed primarily of chitin and additional polysaccharides. Although manual grinding in liquid nitrogen or bead‐beating (with repeated re‐chilling on dry ice) can disrupt these walls, enzymatic digestion has also been proposed (Kenjar et al. 2021). However, its effectiveness may vary by species. A second challenge comes from phenolic compounds, which become increasingly abundant in older (> 1–2 months) axenic cultures (Pizarro et al. 2018). Consequently, younger tissues or fruiting bodies typically yield more DNA with fewer contaminants. Pre‐treatment of mycelia with methanol for at least 2 h, followed by multiple washes, can help remove these phenolics before standard CTAB extraction (Conlon et al. 2021; Cubero and Crespo 2002).

Certain **marine sea slugs** (Sacoglossa) sequester algal chloroplasts (kleptoplasty), introducing potential contaminants that are often undetectable yet detrimental to sequencing. In one example, CTAB extraction from a single individual of the sacoglossan sea slug *Elysia timida* (Risso, 1818) (Männer et al. 2024) initially produced poor sequencing results on a PacBio low‐DNA‐input library. Drawing on the approach of Bein et al. (2025), the standard PacBio polymerase A/B was combined with a high‐fidelity polymerase, KOD Xtreme (Merck, designated as ‘polymerase C’ in Bein et al. 2025), to amplify the sample's DNA by long‐range PCR before library preparation. In early 2025, PacBio announced the addition of polymerase C to their ultra‐low library preparation kit marketed under the Ampli‐Fi trademark. Although amplification poses risks of PCR bias, it offers a viable last resort to obtain cleaner DNA, ultimately enabling a genome assembly with scaffold and contig N50 of 41.8 and 1.92 Mb, far exceeding previously published sacoglossan genomes (Cai et al. 2019; Eastman et al. 2023; Maeda et al. 2021). This underscores the importance of flexible strategies for navigating unexpected contaminants and biochemical idiosyncrasies often encountered in reference genome sequencing projects.

***Take home message:*** it is important to expect pronounced taxon‐specific effects in extraction performance: even established protocols that work for a close relative may require substantial tweaking. Major biochemical obstacles (e.g., phenolics, polysaccharides) should be identified and addressed explicitly with pre‐treatments or alternative enzymes and buffers. It is also valuable to keep a record of successful protocol adaptations and share them (e.g., via protocols.io or WorkflowHub), as these incremental changes are rarely captured in primary papers but are highly relevant for optimisation in other taxa.

## 5 Museomics and High‐Quality Reference Genomes

Although most high‐quality reference genomes rely on freshly collected or optimally preserved material, for some taxa the only feasible option is using existing specimens in natural history collections. In this article, we use ‘museomics’ to refer to the generation of genome‐scale data from museum and archival material (Card et al. 2021; Lalueza‐Fox 2022). These approaches provide a complementary route to taxonomic and temporal coverage, especially for rare, extinct or logistically inaccessible species, and can be integrated into reference genome projects wherever fresh tissue cannot be obtained (Kapun et al. 2025).

Even though natural history collections were never curated with reference genome sequencing in mind, they hold an immense wealth of historical and taxonomic data. Many institutions are now expanding their biobanks to include tissues, cell cultures or DNA suitable for molecular research (Astrin and Schubert 2017; Blom 2021; Card et al. 2021), so that checking for already collected material, rather than planning a new sampling campaign, is increasingly a realistic option (Figure 1). However, for the vast majority of specimens, especially those collected decades or centuries ago, intact nuclei or RNA are rarely preserved, and long DNA fragments may be scarce or absent. Consequently, generating long‐read assemblies from such material is often extremely challenging (collection date > 2000 for animals, Bein et al. 2025), and targeted approaches or short‐read assemblies are usually more feasible than true long‐read reference genomes.

From the perspective of assembly quality, museum and archival material spans a broad gradient of DNA integrity. Recently collected vouchers preserved in ≥ 95% ethanol and stored cold (−20°C to −70°C), or associated frozen tissues and cryobank samples, can still yield long‐read libraries and assemblies that, with low‐ or ultra‐low‐input PacBio HiFi protocols, reach multi‐megabase contig N50 values and approach assemblies from fresh tissue (Bein et al. 2025). By contrast, older dry, fluid‐preserved, or formalin‐fixed specimens typically only support short‐read or targeted‐capture datasets (Campos and Gilbert 2012; Hahn et al. 2024; Ruane and Austin 2017; Straube et al. 2021; Totoiu et al. 2020), which are invaluable for taxonomy, phylogenetics, phylogeography, historical demography and trait evolution but rarely meet current ‘flagship’ reference genome standards for contiguity and completeness.

Some historic specimens are the only surviving examples of extinct or extremely rare taxa, making sequencing of museum material invaluable even when only partial assemblies can be recovered (Davis et al. 2025; Feigin et al. 2018; Manzo et al. 2024; Zedane et al. 2016). In these cases, a high‐quality reference genome is usually generated from a modern sample of the same or a closely related taxon, and short‐read data from museum specimens are then mapped to that reference to derive taxonomic, population, adaptive or functional inferences. Such museomic datasets have clarified how and why some species became extinct (Frei et al. 2023; Vonlanthen et al. 2012), revealed the former diversity within clades, and identified lost lineages whose alleles now contribute to genomic variation in existing populations. For non‐extinct species, however, building a high‐quality reference from fresh tissue is typically more efficient, with museum samples used later for comparative or resequencing studies (Staats et al. 2013).

The degree of DNA degradation in museum samples can vary widely, so that the success of obtaining sufficiently long DNA fragments for reference genome sequencing is unpredictable. In wet collections, DNA quality generally depends on preservation protocols: ethanol‐stored specimens without formalin often yield moderately intact DNA, whereas formalin fixation typically causes extensive fragmentation and chemical modification (Hykin et al. 2015). For dry collections (e.g., herbarium sheets, pinned insects, mammal skins), factors such as heat treatment of herbarium specimens (Bakker 2019), the presence of toxic chemicals (e.g., mercury, arsenic) used historically for pest control (Ballare et al. 2019), or the method of specimen killing in insects (chloroform, ethyl acetate, cyanide) all influence DNA yield and fragment length, often more critically than specimen age. Researchers must also contend with secondary metabolites (e.g., polyphenolics, polysaccharides, pigments, endogenic toxins) that bind or modify DNA (Alsos et al. 2020; Kistler 2012; Kuzmina et al. 2017). Advanced ancient DNA (aDNA) extraction techniques, originally designed for prehistoric remains, can help recover fragmented DNA more effectively and limit contaminants (Gutaker et al. 2017; Marinček et al. 2022), but they are time‐intensive, costly, and often require specialised clean laboratories (Marinček et al. 2022).

In summary, museomics does not replace the need for prospective, high‐quality sampling, but it substantially broadens the taxonomic, temporal, and geographic scope of reference genome initiatives. Where only older or chemically treated specimens exist, the focus shifts to recovering as much reliable genomic information as possible and communicating clearly that these are not ‘flagship’ reference genomes, but nonetheless critical for covering rare, extinct, or otherwise inaccessible taxa.

BOX 4 **Experience: Museomics of Least Weasels.**

This box illustrates how historical museum specimens, combined with a contemporary reference genome, can be used to dissect the genetic basis of adaptive phenotypes and to design monitoring tools, even when high molecular weight DNA is not available.

**Least weasels** ( *Mustela nivalis* L.) across Europe display local adaptation to spatially varying winter snow cover, with morphs manifesting either a white or brown winter coat that enables camouflage on the local background (Mills et al. 2018; Miranda et al. 2021). This colour polymorphism is genetically determined, and a museomics strategy, combined with a contemporary reference genome, pinpointed the melanocortin‐1 receptor (MC1R) gene region as the primary driver of coat‐colour variation (Miranda et al. 2021). To achieve this, researchers sampled 79 dry skin patches from historically preserved specimens and adapted an ancient DNA extraction protocol (Dabney et al. 2013). They tailored each step to accommodate museum DNA's short and fragmented nature. These procedures involved laboratory conditions to avoid contamination, short‐insert library construction, and individual indexing of each specimen's library, ensuring accurate read assignment during multiplex sequencing runs. In mapping these reads to a newly generated draft genome based on a single fresh sample, the team identified specific polymorphisms associated with the observed coat colour morphs. This knowledge enables the design of molecular monitoring tools to track adaptive alleles over time, illustrating how museum specimens, when paired with modern genomic resources, can yield critical insights into both historical adaptation and future evolutionary processes facing climate change (Ferreira et al. 2023).

***Take home message:*** it is important to recognise that for extinct or very rare taxa, short‐read museomics anchored on a modern reference genome can provide powerful insight into adaptation, population history, and lost species diversity. Ancient‐DNA‐style laboratory workflows (including decontamination, short‐insert libraries and individual barcoding) are essential to maximise information from degraded material and avoid cross‐contamination. Even where high‐quality reference genomes cannot be generated from museum specimens, lower‐coverage genomic data such as genome skims or inferred genetic barcodes remain highly valuable as taxonomy‐anchored reference data that support orthogonal species identification in other reference genome projects. These historical genomic resources can then be used to design reduced‐representation approaches (e.g., diagnostic SNP assays) for contemporary monitoring and conservation programmes.

## 6 Collaborative Efforts and Recommendations

Although sequencing technologies continue to progress, generating high‐quality reference genomes remains a challenge. Each step along the way, from securing permits and collecting samples in challenging environments to correctly identifying them to species level, safely shipping them, extracting DNA or RNA, and curating well‐documented vouchers, calls for a specialised skill set and well‐coordinated teamwork. Bringing together conservation officials, local field experts, taxonomic experts, genomicists, sequencing facility staff, museum curators, and biobank managers is often the difference between a successful outcome and a stalled project, even before sequencing. This is particularly true for small, cryptic or rare taxa, where ethical constraints, limited material and/or unresolved or difficult taxonomy make sampling strategies and laboratory workflows substantially more complex than for widely available model species. To support new entrants to large‐scale reference genome initiatives, we provide a practical stepwise checklist from early planning through to data and voucher deposition (Data S1). This checklist is intended as a simple guide to the essential paperwork, preparations, and procedures described in the main text, rather than a replacement for project‐ or taxon‐specific SOPs.

In Europe, ERGA (Mazzoni et al. 2023; McCartney et al. 2024) provides an example of how a collaborative network can streamline the entire process, illustrated by the Biodiversity Genomics Europe project (Böhne et al. 2024; Buzan et al. 2024). Through the pooling of expertise, the establishment of consistent standards, standard operational procedures (SOPs), and the sharing of strategies across multiple disciplines, ERGA provides suggestions on how to overcome problems with cold‐chain management, sample preservation, and permit compliance. Many of these SOPs are made openly available via project‐level repositories, for example the DToL workspace on http://protocols.io for sampling, barcoding and wet‐lab processing, and the BGE/ERGA space on https://workflowhub.eu/sops?page=2, so that practitioners can directly reuse and adapt them in their own projects. Equally important, it connects scientists to each other as well as to existing biobanks and public databases like GGBN or GoaT (Challis et al. 2023), avoiding needless re‐collection and minimising impacts on wild populations. A shared structure reduces duplicate efforts and creates a sense of community ownership over best practices, resulting in continuous improvement and innovation.

Within the Biodiversity Genomics Europe project, where many of these procedures have been piloted at scale, ERGA partners have so far attempted sampling approximately 325 species following the approaches outlined here. Based on internal project summaries, 295 species were successfully shipped and extracted, and by December 2025, 138 have resulted in reference genome assemblies submitted to ENA. Around 30 species were abandoned after repeated extraction failures. These were predominantly taxa from deep‐sea or marine environments, or cases where field conditions made it impossible to maintain a continuous deep‐freeze chain or to implement the full set of recommended sampling steps. This experience suggests that, for most terrestrial, freshwater and near‐shore taxa, the current guidelines enable a relatively high rate of progression from collection to assembly within a three‐year project window. It also highlights specific organismal and logistical contexts where additional methodological development is still needed.

Ultimately, harmonising methods from field sampling to data release amplifies the impact of individual projects, strengthens the biodiversity genomics community as a whole and connects it better with the larger biodiversity science community. The testimonials provided with this article (Box 1, 2, 3, 4) offer only a glimpse of the multitude of approaches, difficulties and solutions needed to sample for reference genome sequencing across countries, biomes and the tree of life. Despite this variation, striving for common guidelines and embracing open exchange of knowledge, for example, through platforms like protocols.io or WorkFlowHub (https://workflowhub.eu) and consortia‐based gatherings, ensures that new developments quickly become part of our collective toolkit.

Looking ahead, sustained cooperation among practitioners, policymakers, and local communities will be critical, particularly as legal frameworks like the Nagoya Protocol or rules for Digital Sequence Information evolve. To ensure that high‐quality, reliable data are consistently delivered by reference genome initiatives, we must highlight achievements, address challenges, and share practical experience in real‐world settings. Ultimately, this will open up new avenues for biodiversity assessment and conservation, population genetics, and evolutionary biology research.

## Author Contributions

A.A.‐T.W., A.B., C.d.G., J.A.L., J.P., K.R., L.L.B., R.A.O. and R.M. contributed to the project conceptualization. A.A.‐T.W., J.P., K.R., L.H., L.L.B., O.V.P., O.Z., P.K.D., R.A.O., S.T. and V.N.L. participated in the investigation. A.A.‐T.W., A.B., A.M.M., A.T., C.B.D.‐N., C.d.G., E.B., J.A.L., J.d.C., J.J.A., J.L.‐F., J.M.‐F., J.P., K.R., L.H., L.L.B., M.P., M.R., P.K.D., R.M., S.T., V.H. and V.N.L. drafted the original manuscript. All co‐authors contributed to the review and editing process, led by C.d.G., K.R., and J.P., J.A.L. and K.R. provided photographs from their research. R.M.W. supervised the work, and C.d.G. managed project administration. C.J.M., C.C., R.M.W., O.V.P., and E.B. secured funding.

## Funding

We acknowledge the support of the Horizon Europe Framework Programme of the European Union under grant agreement no. 101059492. The Biodiversity Genomics Europe (BGE) project is funded by Horizon Europe under the Biodiversity, Circular Economy and Environment (REA.B.3); co‐funded by the Swiss State Secretariat for Education, Research and Innovation (SERI) under contract number 22.00173 and 24.00054; and by the UK Research and Innovation (UKRI) under the Department for Business, Energy and Industrial Strategy's Horizon Europe Guarantee Scheme. J.A.L. was supported by the Spanish Ministerio de Ciencia, Innovación y Universidades, PID2023‐152577NB‐I00. J.M.‐F. was supported by Portuguese Fundação para a Ciência e a Tecnologia under the ERC‐Portugal programme. L.H. was supported by a Johanna Quandt Young Academy fellowship and the German Research Foundation (Project nr: 530763738). L.L.B. was supported by the Ministry of Education, Science and Youth of Sarajevo Canton under contract number 27–02–35‐33087‐6/24 as well as with the financial assistance of the Interreg Euro‐MED Programme, co‐financed by the European Regional Development Fund Community4Nature (Project no. Euro‐MED0300730‐C4Nature). O.V.P. was supported by SciLifeLab and RFI/VR grant from the Swedish Research Council. P.K.D. was supported by the project PID2023‐147388NB‐I00 financed by the MCIN/AEI/10.13039/501100011033/FEDER, UE. R.A.O. was supported by a Natural Sciences and Engineering Research Council of Canada Discovery Grant (RGPIN‐2024‐06892), the New Brunswick Innovation Foundation Talent Recruitment Fund (TRF_2024_007), and a Swedish Research Council Starting Grant (2021–05580). Open Access funding was provided by Freie Universität Berlin.

## Conflicts of Interest

The authors declare no conflicts of interest.

## Supporting information


**Data S1:** men70100‐sup‐0001‐AppendixS1.pdf.

## Data Availability

Data sharing not applicable to this article as no datasets were generated or analysed during the current study. Benefits from this research accrue from the sharing of our cumulated knowledge and experience.

## Endnotes

^1^
Officially recognised by the European Union as Russia's war of aggression against Ukraine (Council of the European Union 2025).
