# Supplementary material for: From Permits to Samples: Addressing Key Challenges for High‐Quality Reference Genome Generation in Europe
Source: Mol Ecol Resour. 2026 Jan 20;26(2):e70100. doi: 10.1111/1755-0998.70100 (PMC12820446; doi:10.1111/1755-0998.70100)
Supplement: Supplementary file 1 — Data S1: men70100‐sup‐0001‐AppendixS1.pdf. [file MEN-26-e70100-s001.pdf]

Supplementary Information

From permits to samples: Addressing key challenges for high-quality reference genome generation in Europe

Katja Reichel, Jaakko Pohjoismäki, Jonas J. Astrin, Astrid Böhne, Chiara Bortoluzzi, Elena Bužan, Javier del Campo, Claudio Ciofi, Camilla B. Di-Nizo, Pradeep K. Divakar, Carola Greve, Vladimír Hampl, Leon Hilgers, Veronika N. Laine, Jennifer A. Leonard, Jesus Lozano-Fernandez, Lada Lukić Bilela, Camila J. Mazzoni, Ann M. McCartney, José Melo-Ferreira, Rita Monteiro, Rebekah A. Oomen, Martina Pavlek, João Pimenta, Michal Rindos, Ole Seehausen, Andrii Tarieiev, Salvatore Tomasello, Olga Vinnere Pettersson, Robert M. Waterhouse, Alexandra A.-T. Weber, Oleksandr Zinenko, Christian de Guttry

Supplementary File S1. Checklist for reference genome permit to sample steps. The table summarizes key stages, questions and responsibilities from early planning to data and voucher deposition, especially for teams joining large consortia.

Stage	Key questions / actions	Who should be involved	When to address
**1. Project scoping**	Define target species and project rationale (conservation, phylogenetic, ecological, bioeconomic).	PI, project team, funders	Proposal / planning phase
	Decide which sequencing data are required (long reads, 3C/Hi-C, RNA, barcodes).	PI, genomicists, sequencing centre	Proposal / planning phase
	Identify in-country partners and taxonomic experts for each target species or group.	PI, national node coordinators, taxonomists	Early planning
	Assess whether existing museum/biobank material could be used, fully or partially, instead of new field sampling.	PI, curators, biobank team, taxonomists	Early planning
**2. Ethics, permits and ABS**	Check if target species or areas fall under CITES, CBD/ABS, Nagoya, regional conventions or specific national legislation.	PI, legal/ABS officer, in-country partners	Early planning, before committing to fieldwork
	Identify competent authorities for species protection, protected areas, and export/import of biological material.	In-country partners, institutional admin support	Early planning
	Clarify ABS and benefit-sharing requirements (PIC, MAT, due-diligence obligations) and document decisions.	PI, in-country partners, ABS focal point	Before any sampling or export
	Identify Indigenous and local community partners where relevant; discuss research aims, data use and non-monetary benefits.	PI, in-country partners, community representatives	Before sampling and well before publication/data release
	Plan how all permit numbers and conditions will be captured in sample and voucher metadata.	PI, data manager, curators	Before fieldwork and before shipping

**3. Sampling design and logistics**	Decide on sampling design (number of individuals, life stages, tissues, replicate sites) for genome and RNA.	PI, genomicists, field team	Early planning
	Choose sampling approach: lethal vs non-lethal, live transport vs field-preserved tissues, opportunistic sampling (e.g. roadkill).	PI, in-country partners, animal ethics committee	Before ethics applications and permits
	Select preservation strategies compatible with target sequencing methods (flash-freezing, dry ice, ethanol, buffers, cell media).	PI, wet-lab team, sequencing centre	Before fieldwork
	Prepare sampling kits, verify access to LN2 / dry ice, labelled/barcoded tubes, PPE, preservatives, coolers/dry shippers, data sheets or apps).	Field team, wet-lab team	Before field campaign
**4. Coordination with collections and biobanks**	Identify suitable museums, herbaria, biobanks or culture collections for future voucher and tissue deposition.	PI, curators, biobank team, taxonomists	Early planning
	Clarify capacity and costs (e.g. accession fees, storage, digitisation) and include them in project budgets where possible.	PI, curators, institutional admin support	Proposal stage or early implementation
	**Contact collections before sampling** to agree on voucher types, tissue formats, shipping conditions and accession procedures.	PI, curators, field team	Before fieldwork or before subsampling existing specimens
**5. Field collection and documentation**	For each specimen, record standardised metadata (date, coordinates, habitat, collectors, permits, method).	Field team, data manager	During collection
	Ensure that at least one physical voucher (or agreed proxy) will be preserved for every genome specimen.	Field team, taxonomists, curators	During and immediately after collection

	Take photographs of specimens (in situ and ex situ) and habitats, with IDs visible where possible.	Field team	During collection
	Minimise handling time before preservation; maintain appropriate temperature for live transport or preserved samples.	Field team	During collection and transport
**6. Species identification**	Plan who will confirm species identity (field expert, museum specialist, barcoding pipeline).	PI, taxonomists, barcoding team	Early planning
	For difficult groups, arrange DNA barcoding (markers, workflows, repositories) alongside morphological identification.	Taxonomists, barcoding team, wet-lab team	During or shortly after sampling
	Ensure that voucher specimens are strongly linked to barcoded tissues and genome samples through shared IDs.	Data manager, curators, sequencing centre	After collection, before sequencing
**7. Vouchering and biobanking**	Prepare specimens according to collection standards (herbarium sheets, pinned insects, skins/ skeletons, cultures).	Field team, curators	Immediately after or during fieldwork
	Deposit vouchers and associated tissues/DNA in agreed museums, biobanks or culture collections; obtain stable identifiers.	PI, curators, biobank team	As soon as practical after sampling and processing
	Where feasible, deposit duplicate vouchers/subsamples in local institutions in the country of origin and in specialist collections.	PI, curators, in-country partners	During accession planning
**8. Sample shipping**	Decide whether to ship whole specimens, tissues, or extracted nucleic acids; minimise the number of shipments.	PI, wet-lab team, sequencing centre	Before preservation

	Verify packaging and labelling for dry-ice or cryogenic shipments (UN1845, Class 9, orientation arrows; inner packaging).	Wet-lab team, couriers	Before shipping
	Prepare customs and regulatory documentation (permits, invoices/declarations, contacts, CN23/HS codes, tax numbers).	PI, institutional admin support, receiving lab/ sequencing centre	Before shipping
	Coordinate shipping dates to avoid weekend/holiday delays and, where possible, arrange dry-ice top-up services.	Wet-lab team, couriers, receiving lab/ sequencing centre	At time of shipment
**9. Laboratory processing – DNA, nuclei, RNA**	Choose tissues with high expected DNA/RNA yield and low inhibitor content for the focal taxa.	Wet-lab team, taxonomists	Before extraction
	Select and test appropriate protocols/kits for HMW DNA, nuclei and RNA; adapt based on pilot extractions.	Wet-lab team, technicians	Early lab phase, before large-scale processing
	Assess DNA quality (purity, concentration, fragment size) and RNA quality (RIN or equivalent) before library preparation.	Wet-lab team, technicians	After extraction
	Document protocol variants and outcomes (tissues, buffers, yields, size distributions) and link to sample IDs.	Wet-lab team, data manager	Throughout lab work
**10. Museomics-specific workflows**	For museum specimens, review preservation history and any chemicals used (e.g. formalin, pesticides, drying/heat treatment).	Curators, wet-lab team	Before subsampling
	Agree with collections on sampling limits and documentation for irreplaceable or type specimens.	PI, curators	Before subsampling

	Decide whether to aim for a de novo assembly or to map reads to a modern reference genome for population/trait analyses.	PI, genomicists	Before library preparation
	For highly degraded DNA, implement ancient-DNA-style protocols (clean labs, short-insert libraries, individual barcodes, strict contamination control).	Wet-lab team, aDNA specialists	During library preparation
**11. Data, metadata and long-term access**	Capture all relevant metadata (specimen, tissue, permits, methods) in a standard manifest.	Data manager, PI, curators	Throughout the project; finalised before genome submission
	Use community standards (e.g. Darwin Core, GGBN, MIxS, ERGA manifest) and broker metadata to INSDC and related portals.	Data manager, sequencing centre	Before and during genome submission
	Ensure that genome records refer to voucher and tissue identifiers, collection institutions, and relevant permit/ABS information.	PI, data manager	At time of genome and metadata submission
	Share SOPs, protocol adaptations and “lessons learned” via open platforms (protocols.io, WorkflowHub, consortium repositories).	PI, wet-lab team, consortium coordinators	After validation of workflows; at project milestones
	Cite identifiers in all publications to maintain traceability of the original material and vouchers	PI, data manager, consortium coordinators	During the publication process
